# Seasonal changes of circulating 25-hydroxyvitamin D correlate with the lower gut microbiome composition in inflammatory bowel disease patients

**DOI:** 10.1038/s41598-020-62811-4

**Published:** 2020-04-07

**Authors:** Katarina Soltys, Martina Stuchlikova, Tibor Hlavaty, Barbora Gaalova, Jaroslav Budis, Juraj Gazdarica, Anna Krajcovicova, Zuzana Zelinkova, Tomas Szemes, Daniel Kuba, Hana Drahovska, Jan Turna, Stanislav Stuchlik

**Affiliations:** 1 Department of Microbiology and Virology, Faculty of Natural Sciences, Comenius University in Bratislava, Ilkovicova 6, 84215 Bratislava, Slovakia; 2 Comenius University Science Park, Comenius University in Bratislava, Ilkovicova 8, 84104 Bratislava, Slovakia; 3 Department of Molecular Biology, Faculty of Natural Sciences, Comenius University in Bratislava, Ilkovicova 6, 84215 Bratislava, Slovakia; 4 National Transplant Organization, Limbova 14, 83303 Bratislava, Slovakia; 5 Department of Internal Medicine, Faculty of Medicine, Division of Gastroenterology, Comenius University in Bratislava and University hospital Bratislava, Ruzinovska 6, 826 06 Bratislava, Slovakia; 6 Department of Computer Science, Faculty of Mathematics, Physics and Informatics, Comenius University in Bratislava, Mlynska dolina F1, 842 48 Bratislava, Slovakia; 7 Department of Gastroenterology, St Michael Hospital, Bratislava, Slovakia

**Keywords:** Metagenomics, Molecular medicine, Microbiome

## Abstract

Higher probability of the development of Crohn’s disease (CD) and ulcerative colitis (UC) as a possible consequence of the north-south gradient has been recently suggested. Living far north or south of the equator is manifested in fluctuation of vitamin D (vitD) levels depending on the season in both healthy and affected individuals. In the present study we investigate the possible link between the seasonal serum vitD level to the microbial composition of the lower gut of Inflammatory Bowel disease (IBD) patients using 16S rRNA sequencing. Decrease of serum vitD level in winter/spring season in a cohort of 35 UC patients and 39 CD patients was confirmed. Low gut microbiota composition of patients with IBD correlated with the serum level of 25(OH)D that directly coupled to seasonal variability of the sunshine in the central European countries. It is supposed to be related to increased abundance of *Actinobacteria* and *Proteobacteria* in UC and *Actinobacteria*, *Fusobacteria*, *Firmicutes* and *Bacteroidetes* in CD. In summer/autumn period, we observed a reduction in abundance of bacterial genera typical for inflammation like *Eggerthella lenta*, *Fusobacterium* spp., *Bacteroides* spp., *Collinsella aerofaciens*, *Helicobacter* spp., *Rhodococcus* spp., *Faecalibacterium prausnitzii*; and increased abundance of *Pediococcus* spp. and *Clostridium* spp. and of *Escherichia*/*Shigella* spp.

## Introduction

Inflammatory bowel disease (IBD) is a modern life-style disease with a worldwide prevalence. IBD involves mainly Crohn’s disease (CD) and ulcerative colitis (UC) the etiology of which differs, so the clinical determination of the disease is difficult. Chronic inflammation of the gastrointestinal tract defined as cycling of the acute inflammation phase and remission is typical for IBD, both CD and UC. While in UC the disease is specifically localized to the colon with different degree of continuous inflammation proximally from the rectum, the CD can manifest anywhere in the gut. To date, there is no clear evidence of a single factor causing the IBD. It is affected by genetic susceptibility, microbiome composition, immune response dysregulation as well as various environmental factors^1^.

The vitD defficiency is common among IBD patients^2,3^ and it has been speculated, that a low level of vitD might be one of the risk factors influencing the IBD^4–6^. The current epidemiological studies of IBD show and upward dynamics in countries distant from the equator^7^, strongly suggesting a correlation between IBD incidence and latitude. This observation is for instance supported by a study from New Zeland that has one of the highest rates of CD in the world^4^. Higher probability of the development of CD and UC^8^ and CD but not UC^9^ as a possible consequence of the latitude-dependent sunlight conditions has been supported by two more epidemiological studies, also reporting that the serum concentrations of vitD levels reached the lowest point after winter^10,11^. The seasonal variability of vitD level correlates with the seasonal pattern of IBD that peaks during the spring season^12–14^. On the other hand, living far north or south of the equator may cause a fluctuation in vitD levels depending on the season (the levels may go down during the winter months due to the lack of sufficient sunlight) also in healthy individuals^15,16^.

The positive effect of the vitD on human health is well known^17,18^, however, its effect on the gastrointestinal microbiome of IBD patients has not been properly elucidated^19^. The human microbiome represents a microecosystem, that is a consequence of mutualism of the host and the bacteria directly influence the health of an individual^1^. The disbalance of the microbiota can lead to dysbiosis of the gut that can mirror also in stool. Bacterial composition is said to play a key role in IBD development^5,20^. Some studies show quantitative reduction of certain bacteria in CD and UC patients compared to healthy individuals^21–23^, in others also qualitative differences in microbiome of the gut and fecal samples likely point to IBD^24–26^. On the contrary some studies reporting no changes in analysed microbiota have been also published^27^. In an open-label study Bashir *et al*.^28^ pointed to the positive effect of vitD supplementation on the microbiome composition in the upper part of the gastrointestinal tract (gastric corpus, antrum, duodenum), but not in terminal ileum, appendiceal orifice, ascending and sigmoid colon and in stools of healthy individuals.

Furthermore, the active metabolite of vitamin D_3_ exerts its regulatory function by binding to the vitD receptor (VDR). Regarding IBD pathogenesis, VDR expression at mRNA and protein levels is significantly decreased in IBD patients^29^. Of the 618 reported variants, the most common SNPs studied regarding various inflammatory based diseases are FokI, ApaI, BsmI and TaqI RFLP polymorphisms the association of that with UC or CD varies between populations. In order to avoid the possibility of the vitD level alterations linked to VDR genetic variability of Slovak population, the association between the most common polymorphisms of VDR and serum vitD level was determined^30^. Since there is still a discussion whether lack of vitD could trigger the microbial changes in IBD a need for a study focused on the bacterial composition characterization in connection with the serum vitD 25(OH)D status and season has emerged. Our group therefore decided to determine the impact of the vitD levels on the bacterial community of adult IBD patients.

In this paper, a metagenomic analysis of ileum and/or colon pinch biopsies from CD (47) and UC (40) patients is presented. To our knowledge, this is the first such study undertaken including samples from both inflamed as well as noninflamed mucosa and stool. The data from the shotgun next-generation sequencing of almost full-length 16S rRNA gene (V1-V6) were used for microbiome alpha-diversity determination and association of vitD and bacterial composition changes, answering the question whether the vitD levels can be related to the microbial composition and consequently the gut homeostasis in IBD patients.

## Material and methods

### Ethical approval

All procedures performed in studies involving human participants were in accordance with the ethical standards of the local ethical committee of the Bratislava Self - Governing region, Slovak republic and with the 1964 Helsinki declaration and its later amendments or comparable ethical standards.

The authors confirm that all methods and experimental protocols were performed in accordance with the relevant guidelines and regulations and were approved by the of the committee of the Ministry of Health of the Slovak republic.

### Informed consent

Individuals were recruited between August 2012 and August 2015 by the IBD center of the Department of Internal Medicine, Division of Gastroenterology and Hepatology, University Hospital Bratislava, Slovak Republic. Informed consent was obtained from all individual participants included in the study. Additional informed consent was obtained from all individual participants for whom identifying information is included in this article.

### Patients and sample collection

All patients, who met the criteria for Caucasian, aged more than 18 and diagnosed UC or CD, were implicated. The heterogenic group of recruited patients involved both genders (male, female), smokers and non-smokers, obese and lean, young and older people. Each patient was characterized by its demographic and clinical status regarding gender, age, duration of the disease, anatomic alternations (fistula, stenosis), surgery, distribution of the disease according to Montreal classification^31^, activity of the disease^32^, serum vitD level and vitD supplementation. In total, 87 patients were included in the study, 40 with an established diagnosis of UC and 47 with CD. Disease activity of individuals was assessed by Mayo score (0–3) for UC and CDAI index (0–352) for CD. 15 patients with active UC (Mayo score 2–3), 25 patients with inactive UC or in remission (Mayo score 0–1) were recruited together with 12 patients with active CD (CDAI > 150) and 35 patients with inactive CD (CDAI ≤ 150). Purposely selected patients for this study represent different clinically significant categories of IBD (Table 1).

**Table 1:** Characteristics of the cohort of patients recruited in the study.

Characteristics		UC (n = 40)	CD (n = 47)
gender	female	17	13
	male	23	34
age (median ± STDEV)		41 ± 13 (23–69)	33 ± 12,4 (20–68)
smoking	smoker	8	9
	non-smoker	32	38
vit.D supplementation	supplemented	5	8
	non-supplemented	35	39
CDAI	≤150		35
	>150		12
MAYO	0–1	25	
	**2–3**	**15**	

Sigmoidoscopy and/or colonoscopy using PENTAX 3885LK conventional white-light colonoscopes were performed in a group of patients. Biopsy and fecal samples were taken from patients undergoing routine examination. 3–5 days before examination the patient was instructed to minimalize the intake of heavily digestive food encompassing seeds, stones etc. The day before colonoscopy laxative agens (Fortrans) was applied. The biopsy sample collection was performed by sigmoidoscopy of mucosa from large intestine (CD, UC) as well as from terminal ileum of the small intestine (CD). Mucosal samples taken from macroscopically inflamed and non-inflamed areas of the gut using standard gape forceps were immediately frozen at −70 °C until further total DNA isolation. The stool samples were collected at home in the evening before the visit of the clinician and stored at −20 °C. The biopsy samples and feces were used for total DNA isolation.

The control group comprised 155 cadaveric organ donors registered in the biobank of National Transplant Organization, Bratislava.

### VitD concentration assessment

Patients were recruited during two seasons of the year, summer/autumn period with high sunlight exposure, from August to October, included and winter-spring period with low sunlight exposure, from February to April, included. These periods were selected according to sunshine conditions in Central Europe; the periods met the criteria of the highest and the lowest expected vitD levels in the serum. Serum vitD, 25(OH)D2 and 25(OH)D3, levels were determined by high-performance liquid chromatography (HPLC, Agilent 1200) under specified conditions (UV detection at 264 nm, flow rate 1 ml/min, temperature 40 °C) for 10 min^11^.

### Tissue and stool sample processing for next-generation library preparation

Total DNA isolation from a single pinch of the mucosa was carried out, after treatment with glass beads and enzymatic lysis, by Qiagen Blood and Tissue kit (Hilden, Germany) according to the manufacturer’s protocol. Briefly, mucosal sample was treated with 140 μl of lysing buffer (20 mM Tris-HCl, 2 mM EDTA, 1.2% Triton-X-100 in distilled water), 30 μl of mutanolysin (600 U), 20 µl of lysozyme and 10 µl of RNase (10 mg/ml). For sample homogenization glass beads (425–600 µm, Sigma) were added. Incubation at 37 °C for 40 min with regular brief vortexing every 10 min was followed by incubation with proteinase K and 200 µl of AL buffer at 56 °C for 1 h and final incubation at 70 °C for 30 min. After addition of 200 µl of absolute ethanol (stored at −20 °C), DNA was purified with QIAamp columns and eluted into 100 µl of ultra-clean millipore water.

For total DNA isolation from fecal samples, stored at −70 °C until use, QIAamp DNA Stool Mini Kit (Qiagen, Germany), was used. Briefly, 180–220 mg of frozen stool samples were homogenized after 1 min of vortexing in 1.4 ml of ASL lysis buffer. After heating to 90 °C for 5 min, vortexing for 15 s and centrifugation for 1 min at 20.817 g, 1.2 ml of supernatants were treated with InhibitEx tablet, vortexed for 1 min and incubated at room temperature for 1 min. Samples were then centrifuged at 20.817 g for 3 min. 200 µl of supernatants were incubated with 15 µl of proteinase K and 200 µl of AL lysis buffer at 70 °C for 10 min. DNA was precipitated with absolute ethanol (stored at −20 °C) and purified with QIAamp column according to manufacturer’s protocol. Concentration and purity of acquired DNA samples were assessed spectrophotometrically by NanoDrop ND-1000 (ThermoFisher Scientific, USA) and Qubit® 2.0 Fluorometer (ThermoFisher Scientific, Waltham, MA USA). Isolates were stored at −70 °C in the DNA bank.

PCR amplification of the 16 S rDNA region (V1–V6) was carried out in a volume of 20 μl using HotStartTaq Plus Master Mix (Qiagen, Germany), 0.5 μM forward primer 27 F (AGAGTTTGATCMTGGCTCAG) and 0.5 μM reverse primer 1062 R (ACAGCCATGCAGCACCT) and 30 ng of template DNA. PCR was performed using the following cycle conditions: an initial denaturation at 95 °C for 5 min, 35 cycles (denaturation at 94 °C for 1 min, annealing at 54 °C for 30 s, extension at 72 °C for 1 min 30 s) and final extension at 72 °C for 10 min. The cleanup of PCR products was carried out by ExoSAP-IT (Affymetrix, Santa Clara, CA) treatment. Final amplicon concentrations were determined using the Qubit Fluorometer (ThermoFisher Scientific, Waltham, MA USA) with Qubit dsDNA HS (High Sensitivity) Assay Kit (Life Technologies, Carlsbad, CA, USA).

Samples were diluted to 2.5 ng/μl in 20 μl volume to meet the requirements in initial step of library preparation. For the sample processing Nextera DNA library kit (Illumina, San Diego, CA, USA) was utilized and the procedure was carried out according to standard library protocol. Briefly, preamplified DNA samples were fragmented by transpozon to app. 300 bp sequences that were purified by DNA Clean & Concentrator™−5 columns (Zymo research, Irvine, CA, USA) and further amplified for 5 cycles by PCR using indexed forward and reverse primers. Size selection of the DNA fragments was carried out by magnetic beads Agencourt® AMPure® XP Reagent beads (Beckman Coulter, Brea, CA, USA). Each final amplicon library was checked for its quality and quantity using Agilent 2200 TapeStation (Agilent Technologies, USA) and Qubit Fluorometer. Amplicon libraries were pooled in equimolar concentration of 4 nM for sequencing run. For sequencing analysis MiSeq Sequencing kit v3 (Illumina, San Diego, CA, USA) was used. Paired end sequencing was carried out with 192 bp read length setting.

### High-throughput data analysis

#### Data preprocessing

Adapters and low-quality ends of sequenced reads were removed using Trimmomatic^33^, based on quality control statistics generated by FastQC^34^. After trimming, only fragments with sufficient length of both reads (>35 bp) were kept for further analysis. Sequences were mapped to the human genome, version hg19 (http://hgdownload.soe.ucsc.edu/goldenPath/hg19/bigZips/), using Bowtie2^35^ with default parameters. Concordantly mapped reads were excluded from the further analysis to eliminate contamination from human tissue. We used prealigned set of 5,181 sequences (http://www.mothur.org/wiki/Silva_reference_files) from Silva database v119^36^ to identify and remove chimeric artefacts. Analysis were performed with tools Mothur^37^ and UCHIME^38^. Chimeric-free sequences were labeled with taxonomy from the full set of the Silva database (153,307 sequences) using Metaxa2 classifier^39^. Overall taxonomic composition of each sample was visualized in form of multi-level pie chart generated by Krona^40^.

### Statistical analysis of metagenomic data

First, only samples matching filtering criteria were kept (for example, samples with the Crohn disease from Stool). Then, the linear discriminant analysis effect size (LEfSe)^41^ tool was used to identify taxons, which are populated differently according to presence of a certain target condition (for example abundance of vitD). In other words, if a taxon is populated significantly higher (or lower) in high vitD environment than in a low vitD environment, it will be identified and marked. The marked taxons were visualized in separate figures and in a cladogram^42^. This analysis was performed for various filtering criteria and target conditions.

ANOSIM is a permutation-based test of the null hypothesis that within-group distances are not significantly smaller than between-group distances^43^. Univariate Mann-Whitney and multivariate Anosim tests were used to assess significance of difference between taxonomic abundances under various conditions. Statistical analysis and visualization were performed using Python and R scripts. Nonparametric measure of rank correlation (Spearman’s rank correlation) was used to determine the statistical dependence between the ranking of two variables.

## Results

### Biological material

Seasonal sample collection of biopsy and stool samples was performed during the two-year period. During the low sunlight period, 42 biopsy samples from patients with UC and 101 samples from patients with CD were obtained. From the high sunlight period 56 biopsy samples from UC and 65 samples from CD patients were available. Based on actual endoscopic findings and the the origin of the biopsy, samples were divided into groups. Samples from CD patients were assessed: 1. inflamed sigma (14), 2. non-inflamed sigma (44), 3. Inflamed terminal ileum (26), 4. Non-inflamed terminal ileum (37). Since UC is exclusively a chronic inflammation of the colon, two groups of samples could be determined: 1. Inflamed sigma (14), 2. Non-inflamed sigma (44). Samples from 8 CD and 5 UC patients supplemented with vitD were excluded from the analysis based on serum vitD level. Stool samples were obtained from 40 UC and 45 CD patients. Additional information on characteristics of patients recruited in the study is included in Table 1.

### VitD and the gut microbiome composition in IBD

**Figure 1:**
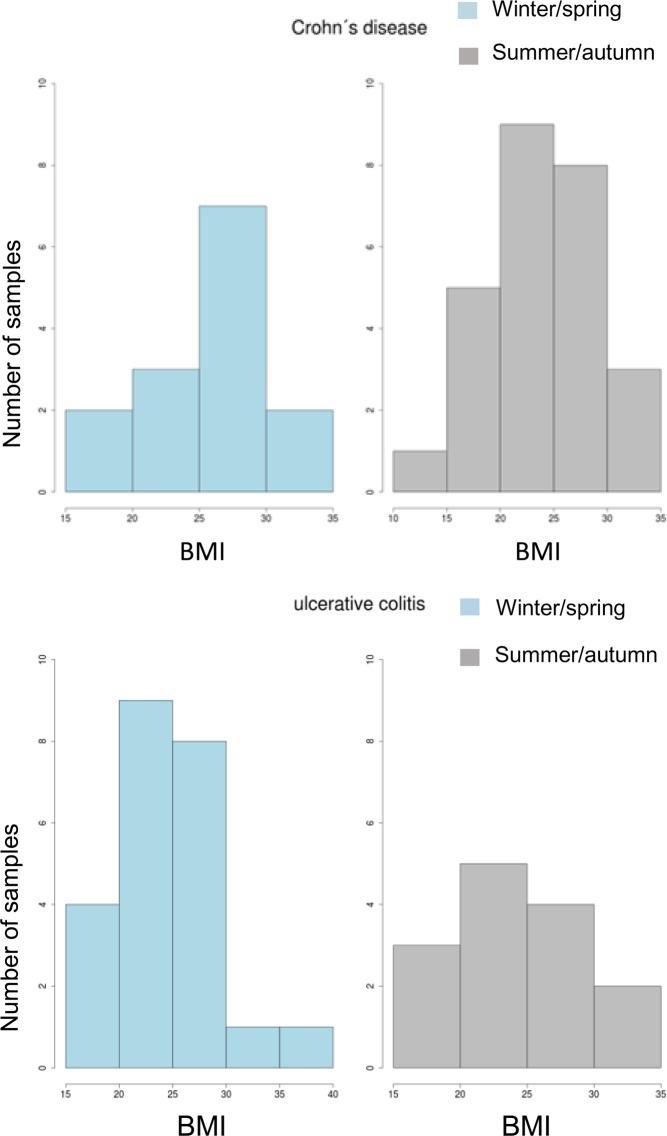
BMI of the cohort of IBD patients involved in the study.

For the microbiome analysis each patient meeting the criteria was included in the study without categorization regarding gender, age or BMI. However, to exclude the possible influence of the BMI status of patients on the microbial composition of the gut in further analysis, we have tested the probability of the significance of distinction of BMI between cohorts of UC and CD patients in winter/spring and summer/autumn. No significant differences could be detected between IBD patients during any season (CD, p = 0.36; UC, p = 0.97) (Fig. 1).

### Ulcerative colitis

#### Correlation of serum vitamin D level with the season

**Figure 2:**
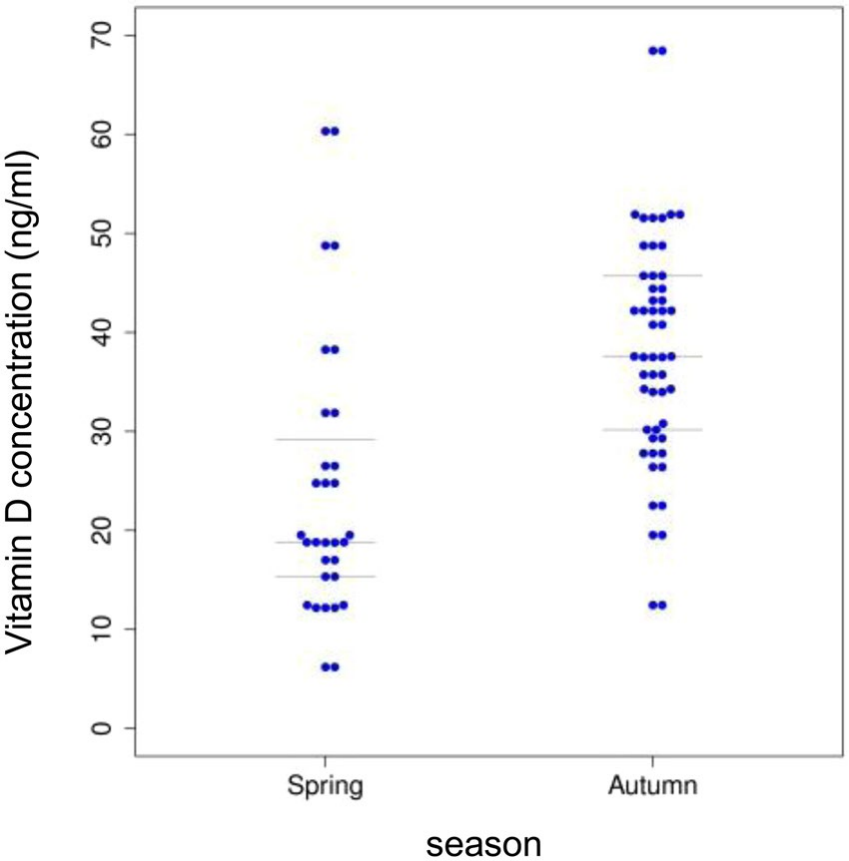
Serum vitD level determined in the cohort of UC patients in relation to the season (winter/spring, summer/autumn).

To determine the seasonal variation of serum vitD level, samples were classified into two seasons: winter/spring and summer/autumn. The serum vitD concentration varied among the seasons, winter/spring (in average 25.05 ng/ml) and summer/autumn period (in average 37.26 ng/ml). Without further categorization of samples according to other variables (gender, smoking, age) during winter/spring season the serum vitD level was significantly lower than in summer/autumn season (p = 7.163e-06) (Fig. 2).

#### VitD level correlation to microbiome of patients with Ulcerative colitis

**Figure 3:**
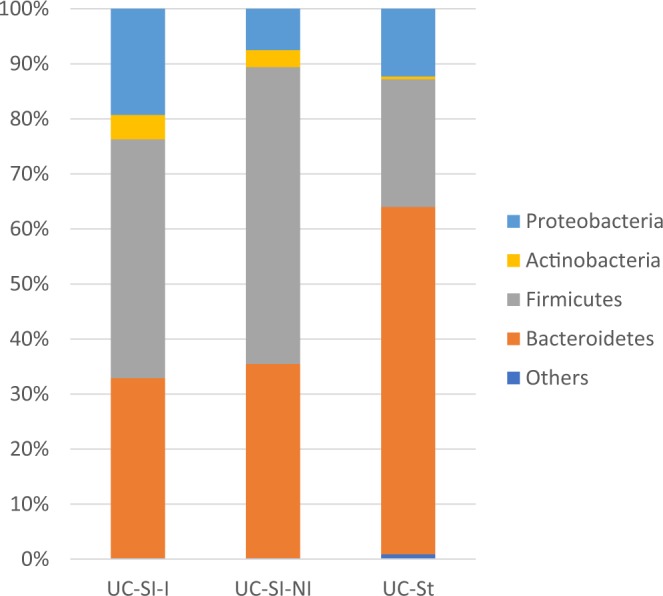
The proportional representation of the most abundant bacterial phyla in human lower gut (sigma) and stool within the cohort of patients with ulcerative colitis involved in the study. UC – ulcerative colitis, Si – sigma, St – stool, I – inflamed, NI –noninflamed.

**Figure 4:**
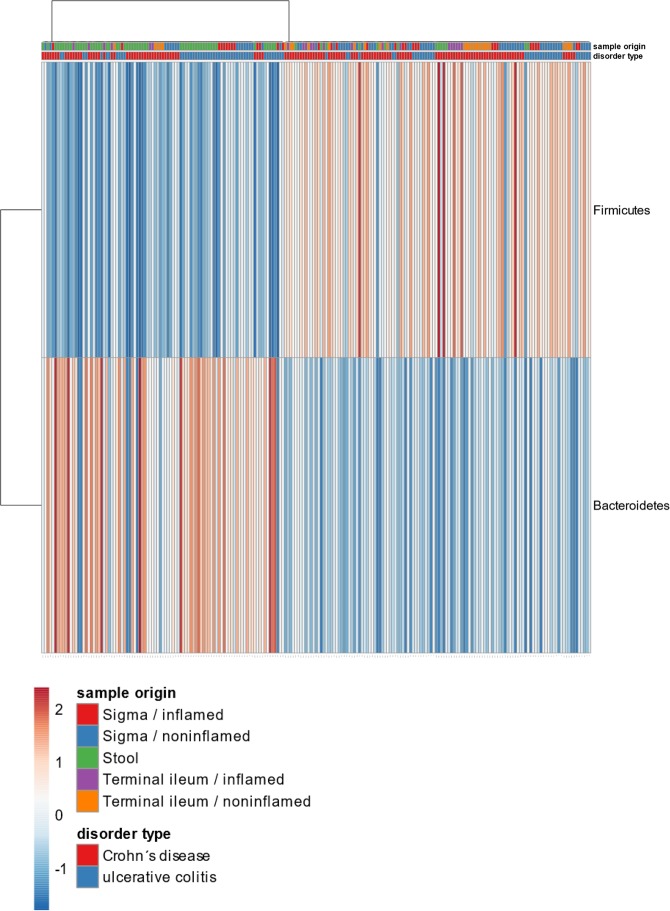
Heatmap visualization of *Firmicutes* and *Bacteroidetes* proportion in mucosal and fecal samples. Almost all stool samples (Crohn’s disease, ulcerative colitis) are dominated by Bacteroidetes, while mucosal samples by Firmicutes.

Regarding the microbiome the dominating phylum of the sigmoid mucosal samples either noninflamed (NI) or inflamed (I) was *Firmicutes* 49,9% (53,9%/43,4%). *Bacteroidetes* represented 34,3% (35,3%/32,7%) and *Proteobacteria* app. 12% (7,5%/19,3%). *Actinobacteria* were the fourth most abundant phylum 3,6% (3,1%/4,4%). The microbiome of stool samples corresponded with mucosal samples in the four most abundant phyla, although the relative amount of *Firmicutes* was lower (23,2%). Here *Bacteroidetes* was the most abundant phylum (63,1%) and *Proteobacteria* (12,3%) and *Actinobacteria* (0,5%) formed the least abundant proportion of the analyzed microbiome (Fig. 3). The observed difference in the proportion of *Firmicutes* and *Bacteroidetes* in mucosal samples and stool was investigated and the diversity of samples was visualized by heatmap (Fig. 4). In most stool samples *Bacteroidetes* were more abundant than *Firmicutes*.

**Figure 5:**
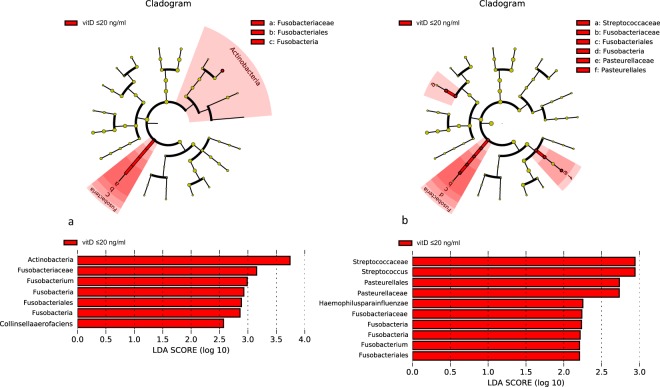
Lefse visualization of significantly altered microbiome taxa of sigmoid tissue of UC patients with low level of serum vitD (≤20 ng/ml; group A). Noninflamed (**a**) and inflamed (**b**) samples are shown. The level of significance ≤0.05 was applied.

Regarding the serum vitD concentration, group A ≤ 20 ng/ml (low), group B 20–30 ng/ml (medium) and group C ≥ 30 ng/ml (high) serum vitD concentration, the microbiome of sigma (both inflamed and noninflamed) and stool was compared. In the cohort of UC patients with low vitD level (≤20 ng/ml) increase of *Fusobacteria*, *Streptococcaceae* and *Pasteurellaceae* represented by *Haemophillus parainfluenzae* was detected (Fig. 5b) in inflamed sigma samples. In noninflamed tissue increase of *Actinobacteria* followed by *Fusobacteria*, especially *Fusobacterium* spp. but also of *Collinsella aerofaciens* was detected, too (Fig. 5a). Microbiome analysis of stool samples haven’t revealed any significant changes.

Exposure to sun has been reported as a factor directly influencing the level of vitD and consequently possibly also the gut microbiome. To assess the influence of the seasonal serum vitD level, mucosal tissue and stool samples collected in winter/spring and summer/autumn period were examined for microbiome composition.

**Figure 6:**
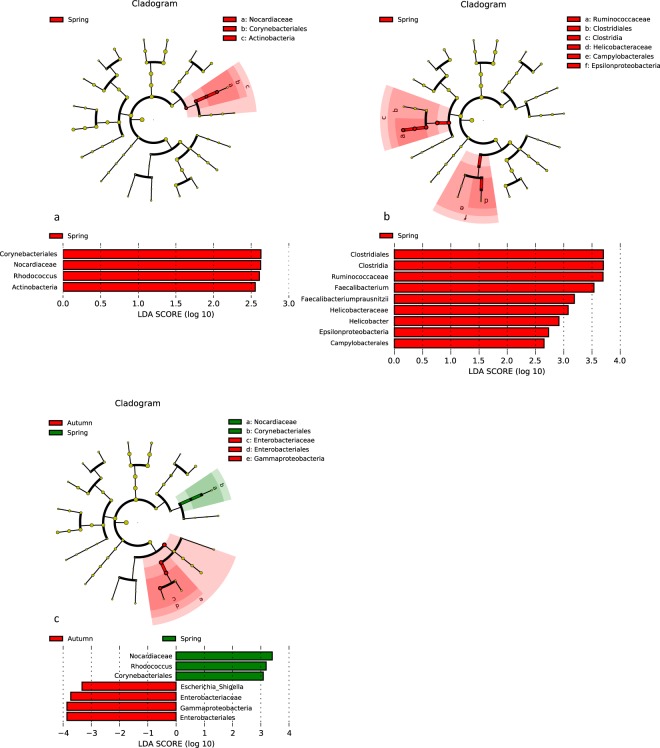
Lefse visualization of significantly altered microbiome taxa of sigmoid tissue of UC patients in winter/spring period in comparison to summer/autumn period. Noninflamed (**a**) and inflamed (**b**) sigma, stool (**c**) samples are shown. The level of significance ≤0.05 was applied.

During the winter/spring period higher level of *Proteobacteria*, esp. *Campylobacteralles* and *Helicobacteraceae* in inflamed sigma (Fig. 6b); but more increased *Actinobacteria*, especially *Corynebacteriales*, *Nocardiaceae* and *Rhodococcus* were assessed in noninflamed sigma (Fig. 6a). The significant changes in the fecal microbiome correlated with the status of the healthy, noninflamed tissue of the patient during the same period. The sequencing of the bacteria from stool samples collected during summer/autumn period showed almost 4-fold increase of *Gammaproteobacteria* represented by *Escherichia*/*Shigella* (Fig. 6c).

To check the microbial composition of the inflamed and the noninflamed analyzed tissue, comparison of two sets of samples regarding their inflammation status and the collection period was carried out. Interestingly, during the winter/spring season, no significant differences in the beta diversity of inflamed and noninflamed samples were detected. However, healthy noninflamed samples from the summer/autumn period showed higher abundance of the representatives of the phylum *Firmicutes* (data not shown).

### Crohn’s disease

#### Correlation of serum vitamin D level with the season

**Figure 7:**
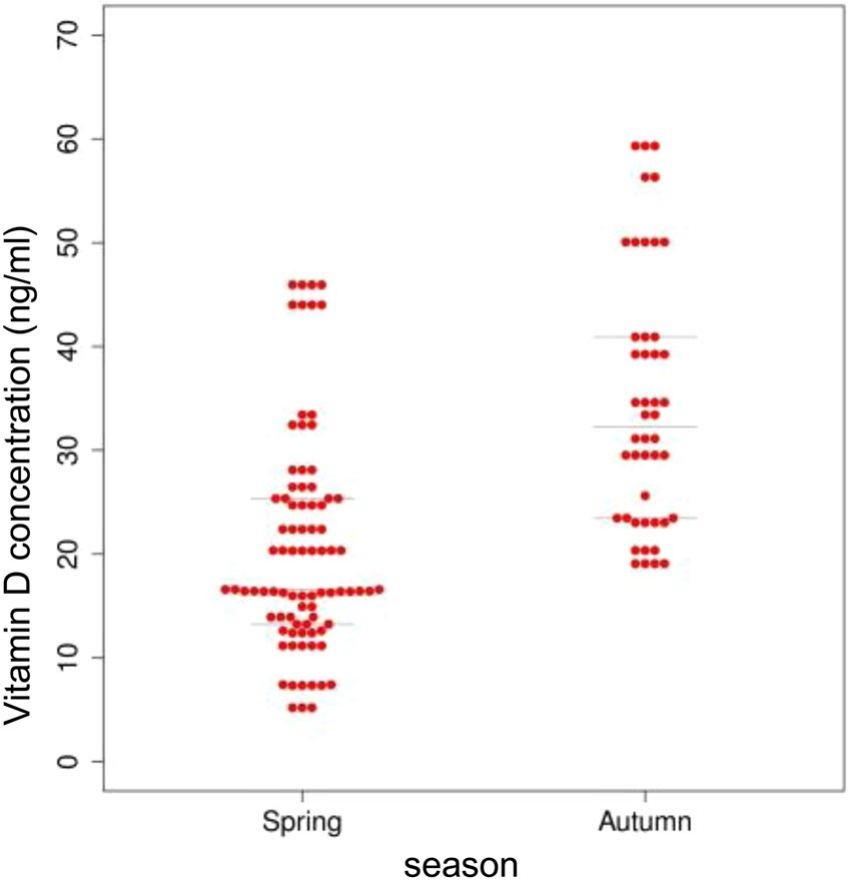
Serum vitD level estimated in the cohort CD patients in relation to the season (winter/spring, summer/autumn).

The seasonal variability of the serum vitD level in blood of CD patients was assessed and significant correlation of the two-season period (winter/spring, summer/autumn) was found. The serum vitD level varied among the seasons, winter/spring (20.23 ng/ml) and summer/autumn period (34.72 ng/ml) significantly (p ≤ 0.05). Without further categorization of samples according to other variables (gender, smoking, age) during winter/spring season the serum vitD level was significantly lower than in summer/autumn season (p = 7.837e-10) (Fig. 7).

#### Analysis of the vitD correlation to microbiome of patients with Crohn’ disease

**Figure 8:**
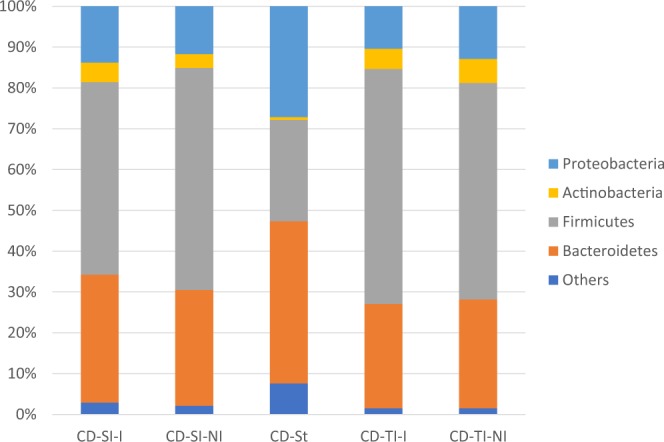
The proportional representation of the most abundant bacterial phyla in human lower gut (sigma, terminal ileum and stool) within the cohort of patients with Crohn’s disease involved in the study. CD – Crohn’s disease, Si – sigma, TI – terminal ileum, St – stool, I – inflamed, NI –noninflamed.

The microbiome analysis of mucosal tissue of the GIT (sigma, terminal ileum) revealed the same profile of main bacterial phyla in both noninflamed and inflamed samples. Presence of the most abundant phylum *Firmicutes* in sigma 52,6% (54,4%/47,2%) and terminal ileum 54,6% (53,1%/57,0%), followed by *Bacteroidetes* in sigma 29,1% (28,4%/31,4%); terminal ileum 26,5% (26,7%/25,3%), *Proteobacteria* in sigma 12,2% (11,7%/13,8%); terminal ileum 11,9% (12,9%/10,3%) and *Actinobacteria* in sigma 3,8% (3,4%/4,8%); terminal ileum 5,5% (5,9%/4,9%). A remarkable difference could be seen between the most abundant phyla of mucosal tissue and stool samples; here *Bacteroidetes* was the most abundant taxon (39,7%), followed by *Firmicutes* (24,8%) and *Proteobacteria* (27,1%) with app. 1:1 ratio. *Actinobacteria* formed only 0,7% of the total amount of analyzed bacteria (Fig. 8).

**Figure 9:**
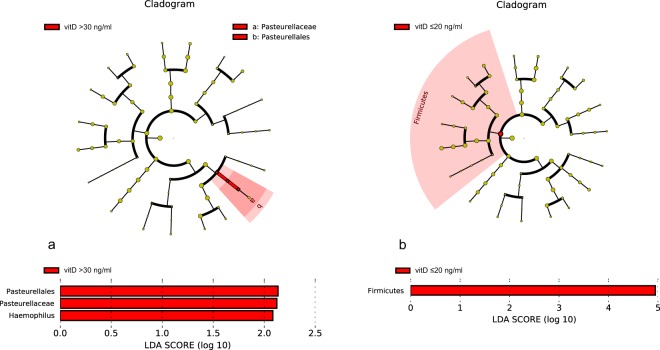
Lefse visualization of significantly altered microbiome taxa of stool (**a**) and inflamed sigmoid tissue (**b**) of patients with Crohn’s disease in relation to vitD level. The level of significance ≤0.05 was applied.

A search for significant change in microbiome possibly correlating with the level of serum vitD was observed as well as by UC patients. The same categorization criteria were applied (Hlavaty *et al*., 2014). The complex microbiome analysis of terminal ileum revealed no significant changes in any inflammation state. However, in the group of patients with low vitD level an increase of *Firmicutes* in the inflamed sigma was observed. On the contrary, high level of vitD significantly correlated with increase of *Haemophilus* spp. in stool (group C) (Fig. 9).

**Figure 10:**
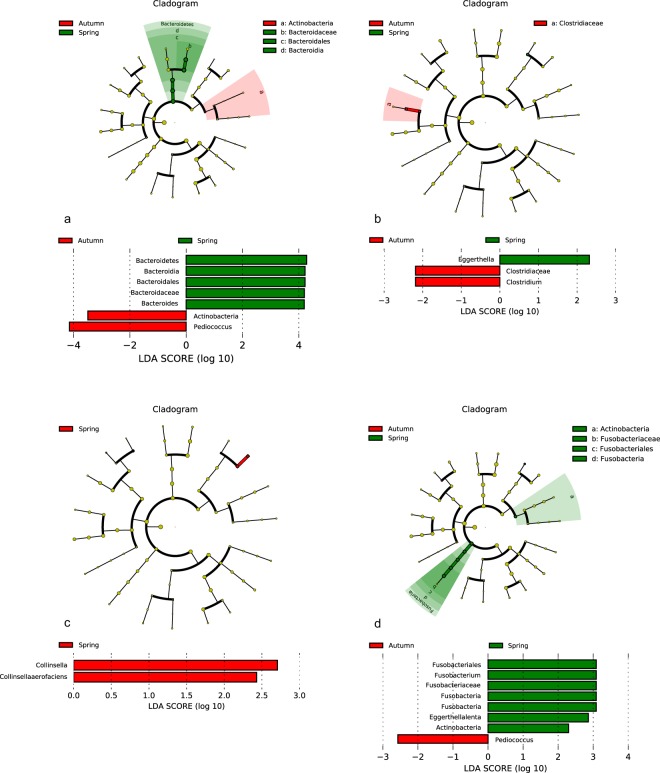
Lefse visualization of significantly altered microbiome taxa of stool (**a**) and mucosal samples of lower gut regarding season. (**a**) stool sample; (**b**) inflamed sigma, (**c**) noninflamed terminal ileum, (**d**) noninflamed sigma of patients with Crohn’s disease. The level of significance ≤0.05 was applied.

Regarding the exposition of individuals to sun the correlation of the seasonal vitD level and the bacterial composition could be observed. In the summer/autumn period significant decrease of *Actinobacteria* (especially *Eggerthella lenta*) in sigma (inflamed and noninflamed) and of *Fusobacteria* (*Fusobacterium*) (noninflamed sigma) was detected. Furthermore, higher abundance of *Clostridium* spp. (inflamed sigmoid tissue) and *Pediococcus* spp. (noninflamed sigma) was detected in lower gut region. On the contrary in stool samples increase of *Actinobacteria* and of *Pediococcus* spp. (*Firmicutes*) accompanied by decrease of *Bacteroides* spp. has been detected. A seasonal decrease in abundance of *Collinsella aerofaciens* in the noninflamed terminal ileum was observed, too (Fig. 10).

## Discussion

Inflammatory bowel disease comprises a group of heterogenous and complex disorders with chronic inflammation. The most common and the most studied are the Crohn’s disease and ulcerative colitis that are known also for their microbial disbalance in the gastrointestinal tract. The positive effect of a therapy using vitD have been proved in several studies. However, little is known about the season-dependent vitD fluctuation in Central European region that can have an impact on the lower gut bacterial composition. In this study we detected the association between the level of serum vitD (25(OH)D2 and 25(OH)D3) in the blood of patients with IBD excluding the possible interventions of the most common SNPs in the VDR receptor and the microbiome alternation of the gastrointestinal tract (GIT). Another important observation was the significant change of seasonal serum vitD level in IBD patients during winter/spring and summer/autumn period that could be related to the bacterial composition of the GIT in IBD patients. In addition, the microbiome composition was followed also in stool were distinct changes of bacterial taxa regarding altered vitD level were found.

Most of the cells in the healthy human body are bacteria of that the dominating phyla are in favor of *Bacteroidetes*, *Firmicutes*^44^ and *Actinobacteria*^45^. However, intestinal inflammation can disturb this balanced community and a reduction of strict anaerobes is accompanied with increase of *Proteobacteria*, mainly *Enterobacteriaceae* family^24^. IBD patients involved in our study possessed the characteristics of typical microbial composition in which the *Firmicutes* and *Bacteroidetes* are dominating taxa, followed by *Proteobacteria* and *Actinobacteria* both in mucosa as well as fecal samples. Whether stool can be used as a reliable marker for gut dysbiosis is still not clear enough. Although the *Firmicutes* to *Bacteroidetes* (F/B) ratio in mucosal samples compared to stool was not significantly altered, vitD level significantly selectively influenced its alfa diversity. Despite several papers showed differences in the composition of the human intestinal and fecal microbiome^44,46,47^, there are studies using the fecal microbiota as a benchmark for the changes of the upper or lower gut microbiome^48^.

Since some studies point to the obesity as an important factor affecting microbiome composition^27^, we investigated its potential influence on our study. Balanced BMI among IBD patients during the two-year period did not indicate any significant shift in the F/B ratio, therefore the obesity could be excluded as a variable with significant impact on our results.

In our study we could confirmed the phenomenon of the seasonality of the vitD level in blood of IBD patients, both CD and UC. During winter/spring season the concentration of serum vitD in UC and CD patients was lower than in summer/autumn period; furthermore, the lack of vitD in CD patients was more severe. Compared to multiethnic study of^49^ Chatu *et al*. in which Caucasians examined from January to June, possessed 41 nmol/L (app. 16 ng/ml) of vitD considered as vitD insufficiency, in the cohort of Slovak Caucasians examined during winter/spring period still higher level of vitD was investigated (CD −20.23 ng/ml, UC 25.05 ng/ml). So far there is one more study of Caucasian IBD population involved in larger cohort of patients^50^ regarding influence of vitD however the vitD level of exclusively Caucasians was not estimated.

The wide positive effect of vitD on human health (cancer, heart disease, diabetes, osteoporosis…) has been extensively investigated and a couple of studies focused on IBD as well. In previous studies positive influence of vitD on UC patients was detected^5,51^, but on the contrary no correlation between the vitD level and the disease activity could be found^52,53^. Our work assessed significant changes in the lower gut microbiome of both UC and CD patients in relation to the seasonal change of sunlight. The key observation in CD cohort is the drop in the level of *Actinobacteria* that is in line with the study on multiple sclerosis^54^. This effect was associated with both, inflamed as well as noninflamed lower gut tissue. Since increased level of *Actinobacteria* in CD patients was established to be associated with the disease^47,55^, obtained results point to the improvement of the gut microbiome. Interesting is the counter status observed within fecal microbiome what indicates that at this stage stool is not suitable mirror of the diseased mucosa status.

In the light of previous study which also reported increased abundance of *Fusobacteriaceae* and decreased level of *Bacteoidales*^56^, the opposite trend observed in our study (decrease of *Fusobacteria* and increase of *Bacteroides* spp.) suggests improvement in the microbiome homeostasis. There are also studies reporting increased^24^ or not significantly altered^48,57^ level of *Bacteroides* in biopsy samples, however, the discrepancy could be explained by various factors: age, diet, disease activity etc.

Patients suffering from UC are commonly vitD deficient^58,59^. According to the results of previous studies^47,55^ increased level of *Actinobacteria* is typical for UC. This observation correlates with the increase of *Rhodococcus* spp., *Helicobacter pylori* and *Campylobacteralles*, that are associated with the dysbiosis in UC^25^ and is a characteristic feature of winter/spring period. The current findings regarding *Escherichia*/*Shigella* group are controversial; according to Pascal *et al*.^60^ it is almost not detected in UC, however certain phylotypes harboring pathogenicity factors *omp*A, *afae* and *USP* belong to more prevalent pathogens in UC^25^. The effect of vitD on the penetration of virulent *E. coli* strains was proved in an *in vitro* study on Caco cells and although it was sufficient to increase clinical and histological parameters of the inflammation, by itself could not sufficiently induce adherent-invasive *Escherichia coli* (AIEC) strain LF82 induced gut injury^61^. In our study no significant changes of *Escherichia* spp. abundance in the sigmoid part of the colon could be observed as well as in healthy volunteers supplemented by vitD^29^, and but 4-fold increase of *Escherichia*/*Shigella* genera in fecal samples in the summer/autumn season was detected. This observation is in accordance with the study of ^62^ in that supplementation of UC patients with vitD lead to no overall fecal microbiome differences, but significant increase of Enterobacteriaceae. It is indicated that differences in microbiome composition and individual taxa abundances are pronounced in fecal and mucosal samples^63^. So far higher abundance of *Escherichia*/*Shigella* in stool has been associated rather with a diet rich in animal proteins and saturated fats^64^, that represents a suitable environment for *Escherichia* spp.^65^ or together with increased *Fusobacterium* as a marker for CD-type IBD^66^. To our knowledge there has been no study describing such an increase of *Escherichia* in fecal samples associated with seasonal increase of vitD.

The effect of the exposure to the sunlight was estimated by significant decrease of abundance of *Actinobacteria* also in CD although in UC only the noninflamed tissue was associated with this change. Furthermore, decrease in *Proteobacteria*, that were proved to be elevated in IBD^48,67^ and more abundant in inflamed UC that inflamed CD^68^ was detected, too. Here also the opposite effect of increase of *Proteobacteria* in stool could be observed. More detailed investigation revealed *Helicobacter* spp. as the only representative of *Epsilonproteobacteria* decreased in tissue samples^69^, while higher abundance of *Escherichia*/*Shigella* spp. (*Gammaproteobacteria*) was detected in stool. Interesting observation regarding decrease in abundance of *Clostridiales*, especially *Faecalibacterium prausnitzii* and *Ruminococcaceae* was associated with the inflamed biopsy of UC patients what correlates with previous study^62^. Reduction of *Faecalibacterium* is set to be primarily associated with ileal CD^70^ and decrease of *Firmicutes* with lower level of vitD^58^. In the study of Machiels *et al*.^21^ with 127 UC patients, also inverted association with the disease activity was detected. However, according to^71^ Prideaux *et al*. ethnicity could be a key factor that might play a role in the *Faecalibacterium dysbiosis*. The influence of the inflammatory status of the tissue in the terms of microbiome changes remains not fully elucidated. Some significant changes in the microbiome composition were assigned to the noninflamed, others to the inflamed part of the intestine. However, there are contradictory findings reporting no significant differences between the status of inflammation within the disease^68^ as well as significant differences in the composition of the mucosal tissue^24^. This could indicate that there must be other factor that selectively influences the microbiome composition, that may influence the vitD accessibility in pathologically altered parts of the intestine.

In our study, lower levels of vitD in winter/spring season are rather associated with more balanced microbiome composition in IBD, higher abundance of *Faecalibacterium prausnitzii* in an intestine and lower level of *Escherichia*/*Schigella* in stool of UC patients. Although in CD higher vitD level was associated with lower proportion of *Actinobacteria* and pathogens like *Eggerthella lenta* and *Fusobacterium* spp., the microbiome composition in winter/spring season was more favorable for lower proportion of *Clostridium* spp., higher proportion of *Clostrida* (*Firmicutes*) in mucosa and increased level of *Bacteroidetes* in stool.

Investigation of the relationship between the serum vitD level and the microbiome composition of IBD patients revealed increased proportion of *Pasteurellales* in fecal samples of patients with hypervitaminosis (vitD over 30 ng/ml) and higher abundance of *Haemophillus* spp. Interestingly, lower level of *Firmicutes* was detected in mucosal samples from inflamed sigma in the cohort of patients with normal vitD level.

In contrast to the previous statements that for patients with CD decrease in *Firmicutes* to *Bacteroidetes* ratio is typical^72^, in our study, the decreased serum vitD level was associated with more favorable microbiome of the gut of IBD patients.

The Human Microbiome Project showed that besides the fact that there is a set of common microorganisms present in human gut, there are also significant intrapopulation and interindividual differences. According to Conlon and Bird^64^ one of the main factors contributing to shifts in the composition and ratios of bacterial taxa could be a diet. Another study correlates high fat diet with increase of *Firmicutes*^73^. However, the results of individual studies are not consistent. The discrepancies might be explained by sample source (biopsy or stool), sampling location (inflammatory or noninflammatory sites), disease activity (active or quiescent), medication, diet, age, smoking, and methods used to analyse the microbiota^74^. Since also our study was focused on the effect of the sunshine period on the microbiome status of UC and CD patients, only patients who visited the IBD center during the selected period (August–October, February–April) were screened. During this period 220 IBD patients (79 UC and 141 CD) visited the IBD center. For 116 IBD patients (141 CD patients and 79 UC patients) the serum vitD level in both seasons was eligible. Since CD as well as UC are very heterogeneous diseases it was very difficult to obtain homogenous groups of patients, even more sets of samples. Many variables considering gender, age, smoking, therapy, supplementation, medication, IBD related surgeries, duration of the disease, disease location and disease behavior have been considered. Finally, 87 IBD patients (47 CD patients and 40 UC patients) were involved, of that samples from 8 CD and 5 UC patients supplemented with vitD were excluded. These are the limits of our study; however, we believe that precise cohort selection can help to clarify and improve the obtained results and emphasize their value.

Nevertheless, the Slovak population of Caucasian origin suffering from IBD, similarly to other geographically distinct populations, show decrease in the level of serum vitD. However, its effect on the gut microbiota composition is more prominent at the genus level differences, rather than at their overall abundancies.

## Data Availability

Results of all analyses are included in this published article. The datasets generated and/or analysed during the current study are available from the corresponding author on reasonable request.
